# Magnolol, a Neolignan-like Drug, Inhibits Porcine Epidemic Diarrhea Virus Replication in Cultured Cells

**DOI:** 10.3390/pathogens12020263

**Published:** 2023-02-06

**Authors:** Xiaoting Wang, Bingqing Chen, Ruisong Yu, Fusheng Si, Chunfang Xie, Zhen Li, Shijuan Dong, Daojing Zhang

**Affiliations:** 1 State Key Laboratory of Bioreactor Engineering, East China University of Science and Technology (ECUST), Shanghai 200237, China; 2 Shanghai Key Laboratory of Agricultural Genetics and Breeding, Shanghai Engineering Research Center of Breeding Pig, Institute of Animal Husbandry and Veterinary Science, Shanghai Academy of Agricultural Sciences (SAAS), Shanghai 201106, China

**Keywords:** porcine epidemic diarrhea virus (PEDV), magnolol, antiviral effect, in vitro

## Abstract

Porcine epidemic diarrhea virus (PEDV) is a destructive pathogen that continues to adversely affect the swine industry worldwide due to a current lack of vaccines and drugs capable of effective disease control. In the present study, the neolignan-like drug, magnolol (MAG), was tested for its ability to inhibit a Vero-cell adapted PEDV strain DR13^att^. Our data revealed that MAG exhibited anti-PEDV activity in vitro, with IC_50_ and CC_50_ values of 28.21 μM and 57.28 μM, respectively. MAG was an efficient inhibitor of viral replication, and repression of viral proliferation was strongest when the host cells were exposed to MAG and the virus at the same time. Although our data indicate that MAG has the potential to be a useful PEDV control agent, in vivo testing of the drug, using animal hosts, is required.

## 1. Introduction

Porcine epidemic diarrhea virus (PEDV), a member of the genus *Alphacoronavirus* in the family *Coronaviridae*, is the causative agent of porcine epidemic diarrhea (PED). The disease is characterized by vomiting, enteritis and severe watery diarrhea, and has a high mortality rate in neonatal piglets [1]. PED outbreaks usually occur in winter and spring, although this seasonal trend has gradually become less defined. PED is endemic or pandemic depending on the viral strain responsible for the outbreak and the immune state of the pig population. A severe outbreak of PED linked to the emergence of the genotype Ⅱ strain occurred in East Asia in 2010, and spread rapidly to Southeast Asia, North America and Western Europe [2,3,4]. PED continues to be a challenge for pig farms in all these areas due to a lack of effective anti-PEDV drugs and the limited success of vaccination programs.

Among the compounds screened for curbing PEDV, those originating from plants have received special attention due to their ready availability and low toxicity. Chemical constituents from the leaves of *Sabia limoniacea* inhibited PEDV replication with a half-maximal inhibitory concentration (IC_50_) value of 7.5–8.0 µM [5], and four oleanane triterpenes impeded PEDV proliferation by downregulating the expression of structural genes [6]. Polysaccharides isolated from *Ginkgo biloba* exocarp prevented PEDV attachment and subsequent entry into Vero cells [7], while aloe extract protected newborn piglets from a lethal challenge of the virus [8]. Recently we reported inhibition of PEDV, both in vivo and in vitro, by three bis-benzylisoquinoline alkaloids [9]. Together, these studies suggest that while plant-derived compounds have considerable potential as PEDV control agents, further extensive screening programs are required.

**Figure 1:**
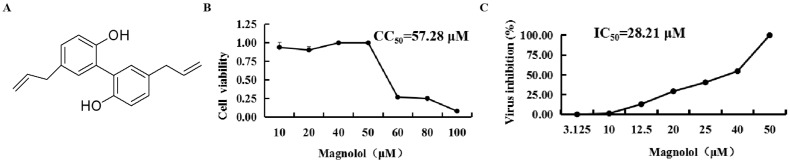
Chemical structure, half-cytotoxic concentration (CC_50_) and IC_50_ of magnolol (MAG)**.** (**A**) Chemical structure of MAG. (**B**) Confluent Vero cell monolayers were cultured for three days in DMEM medium containing two-fold serial dilutions of 100 μM MAG. Cell viability was assessed using the MTT assay, and the CC_50_ values of MAG were calculated. (**C**) Confluent Vero cell monolayers were infected with PEDV DR13^att^, and treated with two-fold serial dilutions of MAG. After three dpi, the viability of the cells in each group was determined using the MTT assay, and the IC_50_ value of MAG was calculated. Data are presented as the mean ± SEM of the three independent experiments.

Magnolol (5, 5′-diallyl-2, 2′-dihydroxybiphenyl, MAG) is a hydroxylated biphenyl-type neolignan, and a major component of the bark of *Magnolia officinalis*, traditionally used in Chinese medicine [10] (Figure 1A). The compound has been assigned anti-oxidation, anti-inflammation, anti-tumor, and antibiotic property [11]. In recent years, this neolignan and honokiol, an isomer of MAG that is also present in *M. officinalis*, have attracted considerable interest as potential antiviral agents [12]. For example, MAG has been reported to inhibit human norovirus surrogates [13], grass carp reovirus (GCRV) [14] and hepatitis B virus (HBV) [15]. MAG and honokiol repressed the proliferation of GCRV by promoting the expression of innate immune-related genes, thereby strengthening the innate immune signaling responses to resist GCRV infection [14]. MAG also inhibited GCRV-induced apoptosis of CIK cells [16], and is reported to modulate SARS-CoV-2 by enhancing the expression of angiotensin-converting enzyme 2 (ACE2), the SARS-CoV-2 receptor [17,18]. To our knowledge, this is the first report describing the inhibition of the attenuated and cell-adapted PEDV strain, DR13, by this well-documented and established plant component.

## 2. Materials and Methods

### 2.1. Cells, Virus and Virus Preparation

Vero CCL-81 cells (African green monkey kidney cells, purchased from ATCC) were cultured in Dulbecco’s Modified Eagle Medium (DMEM) (Hyclone, Logan, UT, USA), supplemented with 10% fetal bovine serum (FBS) (Gibco BRL, Gaithersburg, MD, USA), 100 U/mL of penicillin and 100 μg/mL of streptomycin, at 37 °C and in a 5% CO_2_-enriched atmosphere. The cell-culture-adapted PEDV DR13 strain (JQ023162; isolated from a commercial vaccine of Green Cross, South Korea; DR13^att^) and rPEDV-∆ORF3-GFP (a recombinant PEDV DR13^att^, in which the ORF3 gene in the genome has been replaced by the GFP gene) [19] were propagated in Vero cells with DMEM. Virus titers were determined using the Reed–Muench method, and expressed as tissue culture infective dose 50% (TCID_50_).

### 2.2. Preparation of MAG

Stock solutions of MAG (200 mM) (D&B, Shanghai, China) dissolved in dimethylsulfoxide (DMSO) were prepared and stored at −80 °C. Working solutions were obtained by dilution of the stock with DMEM.

### 2.3. Determination of MAG Cytotoxicity

Aliquots (100 μL) of Vero cell suspensions (2 × 10^4^ cells/mL) were seeded into the wells of 96-well plates and, when the cells had reached 90% confluence (~24 h), the culture medium was discarded and the cells were washed three times with PBS. The confluent monolayers were then exposed to two-fold serial dilutions of a 100 μM MAG solution in DMSO (six wells for each dilution). Vero cell monolayers, and cell monolayers exposed to DMSO solvent without MAG, served as controls. After 3 d incubation (37 °C, 5% CO_2_-enriched atmosphere), cell viability values for each treatment were determined using the MTT assay. Briefly, 20 μL of MTT solution (5 mg/mL) was added to the cell sheet in each well and the well plates were then incubated (37 °C, 5% CO_2_-enriched atmosphere) for 4 h. Spent reaction solution was then removed and 150 μL/well DMSO was added, followed by gentle shaking for 10 min at room temperature. After measuring the optical density of each well at 490 nm, the relative viability of the cells was calculated as follows: cell survival rate (%) = OD (sample)/OD (control) × 100%. The half-toxic concentration (CC_50_) of MAG was calculated using SPSS 21.0 software.

### 2.4. Determination of the Half-Maximal Inhibitory Concentration (IC_50_) of MAG for PEDV

Aliquots (100 μL) of Vero cell suspensions (2 × 10^4^ cells/mL) were seeded into the wells of 96-well plates. When 90% confluence had been attained (~24 h), the cells were infected with PEDV DR13^att^ (multiplicity of infection (MOI) 0.004) and treated with two-fold serial dilutions of MAG (six wells for each dilution). Cell monolayers infected with the virus but not exposed to MAG, untreated cell monolayers, and monolayers exposed only to DMSO served as controls. Well-plates were incubated for 3 d (37 °C, 5% CO_2_-enriched atmosphere) following virus infection, and cell viability levels after each treatment were determined using the MTT assay. Relative virus inhibition rates of MAG were calculated as follows: inhibitory rate of the compound for the virus (%) = (OD (sample) − OD (virus control))/(OD (cell control) − OD (virus control)) × 100%. The half-maximal inhibitory concentration (IC_50_) of MAG was calculated using SPSS 21.0 software.

### 2.5. Immunofluorescence Assay (IFA) of PEDV-Infected Vero Cells

PEDV-infected Vero cells were washed twice with PBS and fixed with 4% paraformaldehyde for 15 min. Cell membranes were made permeable by treating with 0.1% Triton X-100 in PBS for 15 min at room temperature. After blocking with 5% bovine serum albumin (BSA), cells were stained with anti-PEDV M polyclonal antibody for 1 h, rinsed three times with PBS, and then incubated with Alexa Fluor 488-conjugated goat anti-rabbit antibody (Beyotime, Shanghai, China). Nuclei were visualized using DAPI nuclear staining, and pictures of fluorescent cells were captured using an EVOS fluorescence microscope (M7000, Thermo Fisher Scientific, Waltham, MA, USA) at 100× magnification.

### 2.6. Assay of PEDV Inhibition by MAG Using Virus Replication Markers

MAG inhibition of PEDV was investigated by measuring the level of PEDV M protein expression in infected cells, and by determining viral titers and PEDV mRNA levels according to the following protocols:

Vero cells were seeded into 48-well plates (4 × 10^4^ cells/well). After 24 h culture, the cells were treated with 30 μM MAG for 1 h and then inoculated with PEDV DR13^att^ (MOI 0.1). Spent reaction solutions were removed after 2 h incubation (37 °C, 5% CO_2_-enriched atmosphere) and fresh maintenance medium containing 30 μM MAG was added. Cells were fixed at 24 h post infection (hpi) and subjected to immunofluorescence staining.

Vero cells were seeded into six-well plates (4 × 10^5^ cells/well), cultured for 24 h, and then inoculated with PEDV DR13^att^ (MOI 0.1). Inocula were removed after 2 h incubation and fresh maintenance medium containing 30 μM MAG was added to the wells. Supernatants were collected at 36 hpi and assayed for PEDV titers and M protein mRNA levels.

### 2.7. Effect of MAG Application Time on PEDV Proliferation

**Figure 2:**
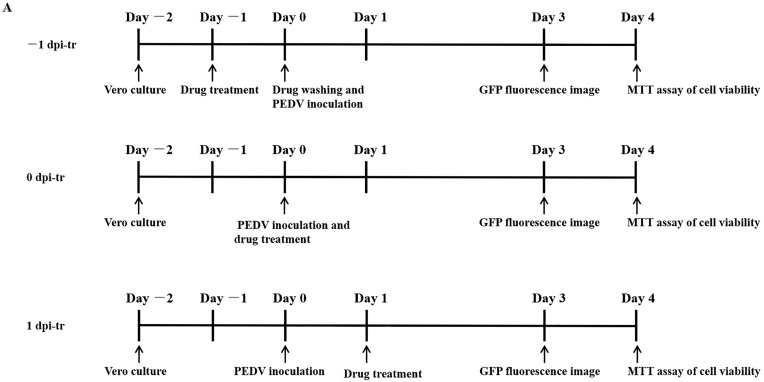

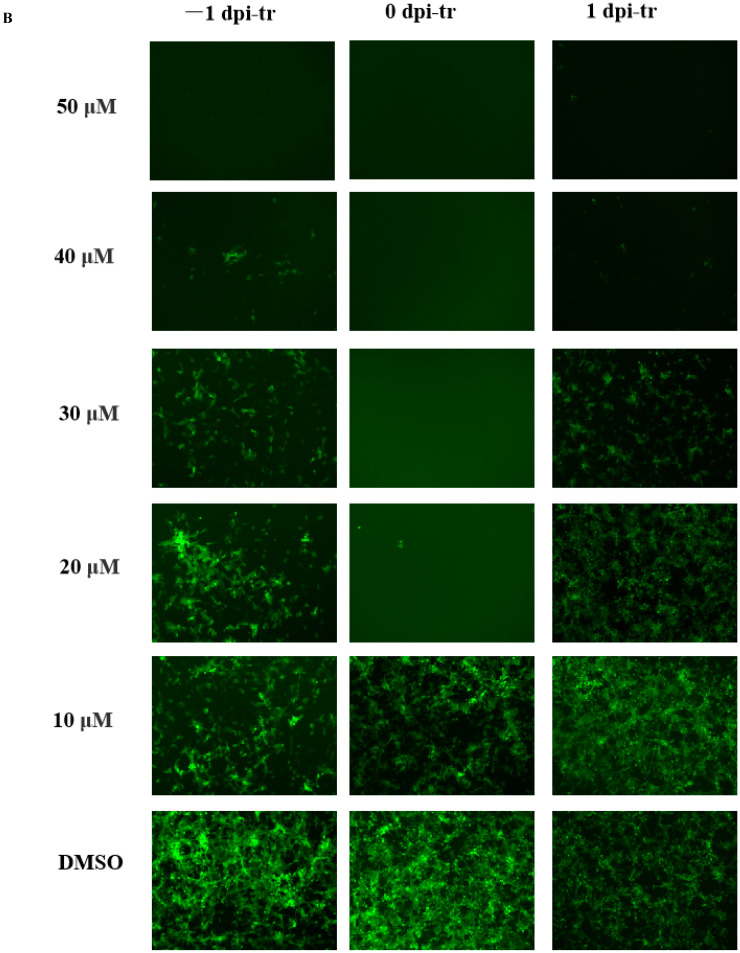

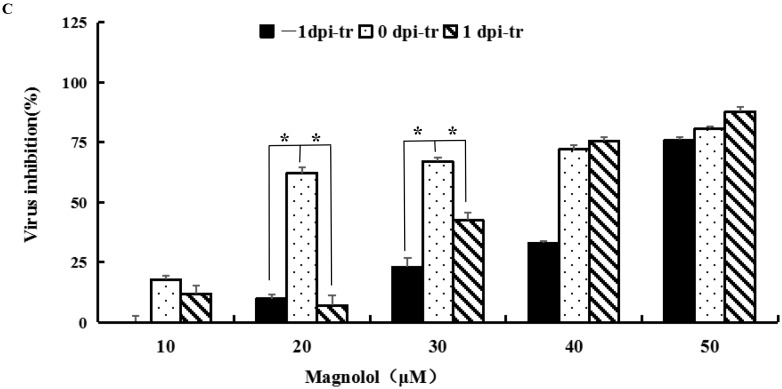
Dose- and time-dependent antiviral activities of MAG. Vero cells were infected with rPEDV-∆ORF3-GFP together with MAG at the concentrations shown, or with the vehicle (DMSO) at a final concentration of 0.05%. MAG was added 24 h before, during, or 24 h after infection of the Vero cells with PEDV. (**A**) Chronology of MAG treatments and assay operations. (**B**) GFP expression images of rPEDV-∆ORF3-GFP for different treatments as indicated. (**C**) Rates at which the different treatments inhibited virus replication. Data are presented as the means ± SEM of the three independent experiments (* *p* ˂ 0.05).

To determine if PEDV proliferation rates were influenced by pre- and post-exposure of Vero cells to MAG, relative to the time at which the cells were subjected to viral challenge, MAG was applied to Vero cells 1 d before, immediately after, or 1 d after exposure to the virus according to the following protocols:

Vero cells were seeded to 96-well plates (2 × 10^4^ cells/well) and incubated (37 °C, 5% CO_2_-enriched atmosphere) for 24 h before adding MAG (−1 dpi-tr) at concentrations of 10, 20, 30, 40 and 50 μM. After a further 24 h incubation, the medium containing MAG was removed and the wells washed three times with PBS. Cells were then infected with rPEDV-∆ORF3-GFP (MOI 0.01) and incubated for 2 h (37 °C, 5% CO_2_-enriched atmosphere) to allow the virus particles to combine with receptors on the cell surface. Any unbound virus was then removed, the infected cell layers were washed and immersed in fresh medium, and the well-plates were incubated for a further 3 days. Alternatively, MAG was applied to the Vero cells immediately after (0 dpi-tr), or 24 h after (1 dpi-tr) infection with the virus (Figure 2A), until the test was terminated. All the samples were subjected to GFP fluorescence imaging 3 dpi, and virus inhibition rates were assessed 4 dpi using the MTT assay.

### 2.8. Effect of MAG on Different Phases of the PEDV Replication Cycle

**Figure 3:**
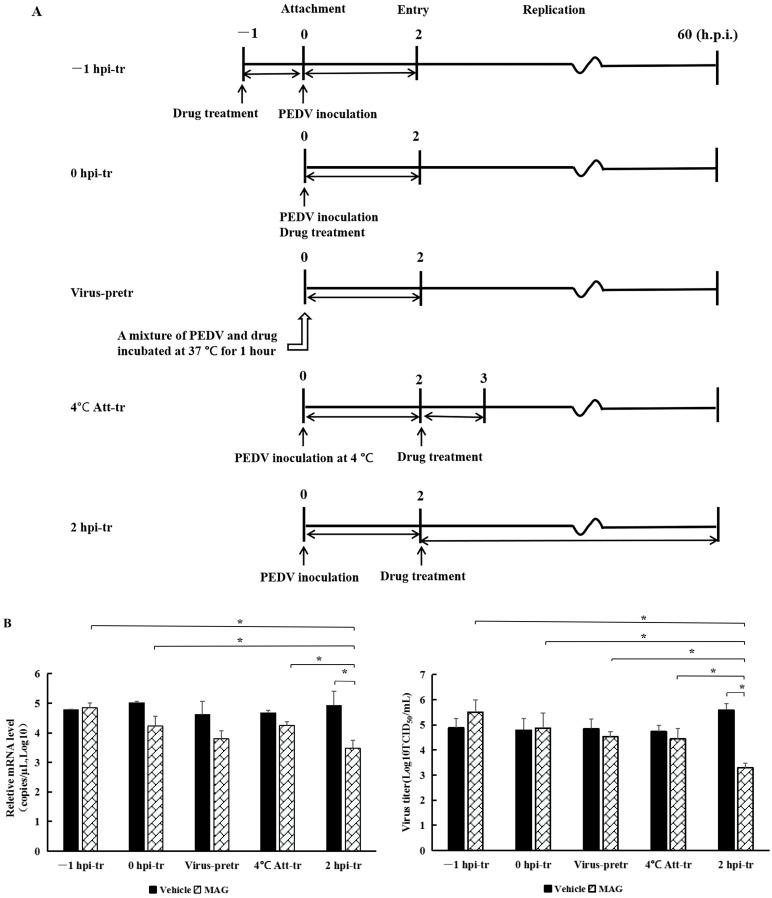

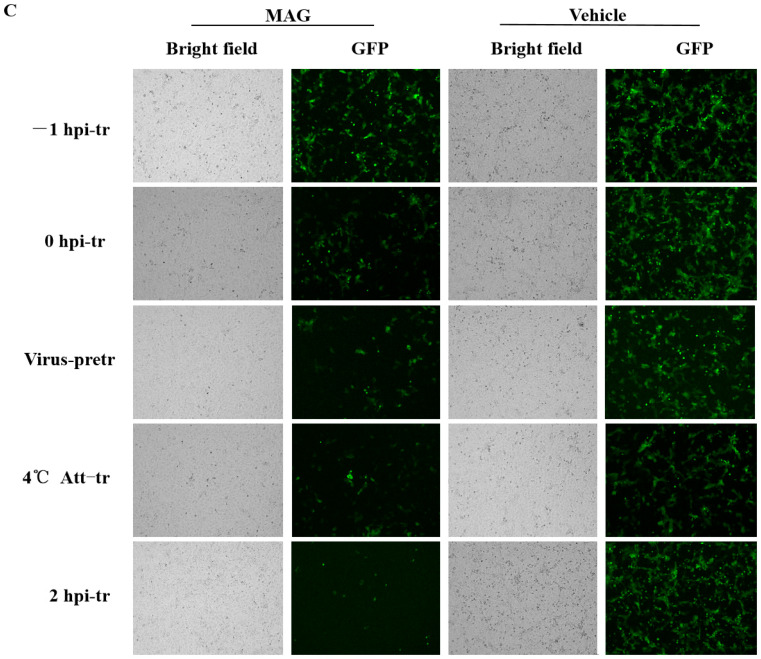
rPEDV-∆ORF3-GFP titers, M protein mRNA levels and GFP expression images after MAG treatment at different stages of the virus life cycle. (**A**) Chronology of MAG treatments and assay operations. For the sake of clarity, only the attachment, entry and replication stages of the virus life cycle are shown. In −1 hpi-tr samples, cells were treated with MAG 1 h before infection with virus. In the 0 hpi-tr samples, MAG was applied to the cells at the same time as the virus. In virus-pretr samples, the reaction components were first incubated at 37 °C for 1 h before adding to the cells. In 4 °C Att-tr samples, viruses were first incubated with Vero cells at 4 °C for 2 h. The inoculum was then removed and the temperature was raised to 37 °C prior to drug treatments and further incubation. In 2 hpi-tr samples, MAG was applied 2 h after virus infection. (**B**) 60 hpi titrations and expression of rPEDV-∆ORF3-GFP M protein mRNA using different treatment protocols. Data are presented as the mean ± SEM of the three independent experiments (* *p* < 0.05). (**C**) GFP expression images of rPEDV-∆ORF3-GFP using different treatment protocols as indicated.

To investigate the effects of MAG on different phases of the PEDV replication cycle, the wells of six-well plates were seeded with Vero cells (4 × 10^5^ cells/well), incubated (37 °C, 5% CO_2_-enriched atmosphere) to 90% confluence, and treated with MAG as follows: (Figure 3A).

Treatment 1 (−1 hpi-tr): Cells were pretreated with MAG (30 μM) at 37 °C for 1 h. The medium containing MAG was then removed and the Vero cells were washed twice with PBS and infected with rPEDV-∆ORF3-GFP (MOI 0.01). Following incubation for 2 h at 37 °C, the inoculum was removed, the cells were washed twice with PBS and, after adding fresh DMEM, incubated as above for a further 60 h before assessment.

Treatment 2 (0 hpi-tr): freshly prepared inoculum from MAG (30 μM) and rPEDV-∆ORF3-GFP was applied to Vero cells (MOI 0.01). Following 2 h incubation at 37 °C, samples were subjected to further work-up as described for Treatment 1.

Treatment 3 (Virus-pretr): MAG and the recombinant virus rPEDV-∆ ORF3-GFP was preincubated together for 1.0 h at 37 °C and then used to inoculate Vero cell monolayers. After 2 h incubation at 37 °C, samples were subjected to further work-up as described for Treatment 1.

Treatment 4 (4 °C Att-tr): Vero cells were inoculated with rPEDV-∆ORF3-GFP at 4 °C for 2 h. The inoculum was removed, the cells were washed twice with PBS and incubated with MAG (30 μM) for 1 h at 37 °C. MAG was then removed and, after adding fresh DMEM, incubated as above for a further 60 h before assessment.

Treatment 5 (2 hpi-tr): The protocol was the same as 4 °C Att-tr, except that the inoculation with rPEDV-∆ORF3-GFP was carried out at 37 °C and MAG remained in the culture system until the test was terminated 60 hpi. Virus titers and M protein mRNA levels in the culture supernatants were determined. Cell imaging was undertaken using a Zeiss Scope A1 microscope (Zeiss Microsytems, Oberkoheng, Germany).

### 2.9. PEDV RNA Extraction and Real-Time RT-PCR

Total RNA was extracted from cells using the TIANamp virus RNA kit (Tiangen, Beijing, China) and subjected to reverse transcription with a reverse transcription reagent (Promega, Fitchburg, WI, USA). Standards for SYBR Green real-time RT-PCR were produced by cloning the 486 bp membrane (M) gene fragment into the pJET1.2/blunt vector (Thermo Fisher Scientific, USA) to construct recombinant plasmids. Primers for standards (sense: 5′-TATTCCCGTTGATGAGGT-3′; antisense: 5′-GCAACCTTATAGCCCTCT-3′) and for qPCR (sense: 5′-TCTTGTGTTGGCACTGTCAC-3′; antisense: 5′-TGCAAGCCATAAGGATGCTG-3′) were synthesized by the Sangon Company (Shanghai, China). Real-time RT-PCR was performed using an ABI 7500-fast Real-time PCR system (ABI, Los Angeles, CA, USA). Each 20 μL qPCR reaction contained 2 μL reverse transcription sample, 10 μL TliRNaseH Plus (2×), 0.4 μL forward and reverse primers (10 μM), 0.4 μL ROX Reference Dye Ⅱ (50×), and 6.8 μL sterile, purified water. Amplification conditions were: 95 °C for 30 s, followed by 40 cycles at 95 °C for 5 s and 60 °C for 34 s. All samples and standards were carried out in triplicate. Tenfold serial dilutions of the PEDV standards (recombinant plasmids) were used to perform qPCR and establish a standard curve. Concentration of sample was calculated by plotting Ct values against the standard curve established by serial dilutions of PEDV standards from 10^1^ to 10^8^ copies/μL.

### 2.10. Statistical Analysis

Statistical analyses (one-way ANOVA) were performed using SPSS software (SPSS 21.0 for windows). All assays were conducted at least in triplicate. Data are expressed as mean values ± standard error (SEM). The *t*-test was employed to determine the statistical significance (*p* < 0.05) of the differences between the means. Data relating to viral RNA copies and virus titers were converted to log10 to maintain a normal distribution.

## 3. Results

### 3.1. Cytotoxicity and Anti-PEDV Activity of MAG

The half-toxic concentration (CC_50_) of MAG for Vero cells was 57.28 μM (Figure 1B). Cytopathic effects (CPE) were obvious 3 dpi in the vehicle-treated (0.025% DMSO) cells, but cells treated with the highest dilution of MAG remained morphologically unchanged. Alleviation of the CPEs observed in cells infected with PEDV DR13^att^ prior to treatment with different concentrations of MAG was dose-dependent. The IC_50_ value for MAG was calculated to be 28.21 μM (Figure 1C), suggesting that the compound was potentially anti-PEDV.

### 3.2. MAG Inhibits PEDV Infection and Proliferation in Vero Cells

**Figure 4:**
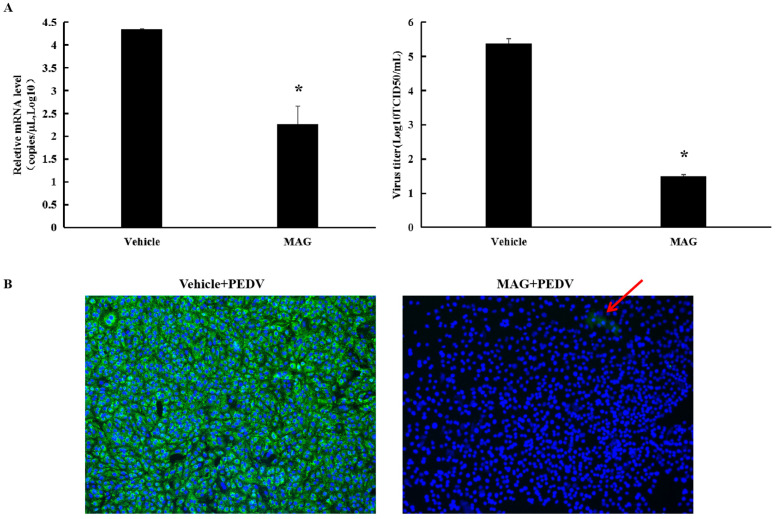
MAG inhibition of PEDV replication in vitro. (**A**) Vero cells were infected with PEDV for 2 h, after which the inoculum was removed and fresh maintenance medium containing 30 μM MAG was added. Supernatants were collected 36 hpi, and virus titers (TCID_50_) and PEDV mRNA levels were determined. Vehicle controls consisted of DMSO at a final concentration of 0.015%. Data are presented as the mean ± SEM of the three independent experiments (* *p* ˂ 0.05). (**B**) Vero cells were treated with 30 μM MAG for 1 h and then infected with PEDV DR13^att^. The inoculum was removed after 2 h incubation and fresh maintenance medium containing 30 μM MAG was added. Vero cells were fixed and subjected to immunofluorescence staining using anti-PEDV M polyclonal antibody 24 hpi. Red arrow indicates the rear immunofluorescence staining of PEDV M protein (green) in MAG+PEDV treated cells.

As shown in Figure 2A, 30 μM MAG significantly decreased PEDV M protein mRNA levels and viral titers in Vero cells compared to vehicle-treated controls. IFA revealed little PEDV infection in cells following treatment with 30 μM MAG (Figure 4B), suggesting that the compound was able to block PEDV infection and/or proliferation in vitro.

### 3.3. Dose- and Time-Dependent Antiviral Activities of MAG

Vero cells were treated with different concentrations of MAG (i) 24 h before infection with PEDV(−1 dpi), (ii) immediately after infection (0 dpi), and (iii) 24 hr after infection (1 dpi), to determine if the time at which cells were exposed to MAG relative to the initiation of virus infection affected the anti-PEDV function of MAG. GFP fluorescence imaging revealed that treatment of cells with 20 μM and 30 μM MAG inhibited virus proliferation when MAG was applied to the Vero cells immediately after inoculation with PEDV. Viral titers were measured 3 dpi (Figure 2A). As shown in Figure 2B, inhibition of virus replication was more pronounced at higher concentrations of MAG, irrespective of which protocol was adopted. GFP fluorescence imaging revealed that 20 μM and 30 μM MAG inhibited virus proliferation more strongly when applied at the same time as PEDV infection compared to the pre- and post-treatment protocols. Virus inhibition was observed at 20 μM concentrations of MAG in the co-treatment tests, but not in the pre- and post-treatments, where an inhibitory response was detected only when the cells were treated with higher MAG concentrations (Figure 2C). When 50 μM MAG concentrations were used, all three treatment protocols resulted in similar virus inhibition rates. These data indicate that MAG is a more efficient inhibitor when employed at the same time as the host cells were infected with PEDV.

### 3.4. MAG Inhibits PEDV by Interfering with the Replication Process

MAG inhibition of PEDV was the strongest when host cells were infected with the virus and treated with MAG at the same time. In order to determine which phase of the virus replication cycle was implicated, we adopted the following protocols (Figure 3A): −1 hpi-tr: attachment; 0 hpi-tr: attachment and entry; virus-pretr: interaction of MAG and virus; 4 °C Att-tr: entry; 2 hpi-tr: virus replication. Significantly lower PEDV M protein mRNA levels and viral titers at 2 hpi-tr compared to the virus infection control (Figure 3B) suggested that MAG was most effective at the viral replication phase. Virus titers at 2 hpi-tr were significantly lower (*p* < 0.05) compared to values recorded for the other four protocols, and protein mRNA levels were significantly lower (*p* < 0.05), except in the case of the virus-pretr protocol. Fewer GFP fluorescence signals were also recorded with cells treated according to the 2 hpi-tr protocol (Figure 3C). We conclude from these data that MAG is an efficient inhibitor of PEDV replication in cultured cells.

## 4. Discussion

Magnolol belongs to the neolignan group of compounds that have been reported to exhibit anticancer, anti-inflammatory [20] and antifungal effects [21], as well as antiviral activity affecting noroviruses, HBV and GCRV [13,14,15]. To our knowledge, there have been no confirmed reports of anti-coronavirus activity associated with MAG so far, although honokiol, an isomer of MAG, has been shown to inhibit SARS-CoV-2 [22]. However, we now report that the treatment of Vero cells with MAG (20 μM and 30 μM), at the same time as they were infected with PEDV, was highly effective in blocking virus proliferation in vitro. Inhibition of virus replication was not observed with 20 μM and 30 μM concentrations of MAG when experiments were conducted using pre-treatment and post-treatment protocols. This may be due to an inability of MAG to bind with virus receptors on the host-cell surface, thereby blocking the entry of the virus. A recombinant PEDV strain of DR13^att^ attained 10^6^ TCID_50_/mL 24 hpi [19], thus exposure to MAG 1 dpi was clearly too late to inhibit viral proliferation. MAG was found to be an effective inhibitor only when it was applied shortly after post-virus infection.

Different drugs employ different strategies in order to block an attack by pathogens. For example, honokiol inhibits SARS-CoV-2 by partial inhibition of furin-like enzymatic activity [22]. However, although honokiol and MAG are closely related both in terms of origin and structure, no such activity has been detected in the latter. However, we have reported that bis-benzylisoquinoline alkaloids inhibited PEDV propagation by denying the virus access to host cells [9]. In a similar study using SARS-CoV-2, bis-benzylisoquinoline alkaloids blocked host calcium channels, thereby inhibiting Ca^2+^-mediated cell fusion, and suppressing viral entry [23]. In the present study, while MAG was found to inhibit PEDV replication, we found no evidence that other phases of viral reproduction, including adsorption and entry, were involved. Additionally, we did not observe any compounding of the drug to the virus. These results are similar to those recorded in a study determining the effects of MAG on *Micropterus salmoides* rhabdovirus (MSRV). In this case, hydrogenated magnolol (magnolol derivatives) had no effect on MSRV adsorption or infectivity but assumed an inhibitory role 6–8 h into the virus replication phase [24]. Further study is required to identify more precisely the nature of the MAG-sensitive stage in the PEDV reproduction process.

IC_50_ value and the selective index (SI) value are used to evaluate the potential of a particular drug application. In this study, the IC_50_ value for the anti-PEDV activity of MAG in vitro was calculated to be 28.21 μM. An earlier study involving MAG and MSRV quoted an IC_50_ value of 19.06 µM [24]. Unfortunately, the CC_50_/IC_50_ value (SI) of MAG for PEDV was only 2.03, well below the values of other compounds of botanical origin, e.g., cepharanthine (CEP, 11.83), tetrandrine (TET, 7.08), and fangchinoline (FAN, 4.51) [9]. However, although a low SI index is generally considered to be a negative feature when assessing the potential of a drug, other factors should be taken into account. These include the route of administration, the metabolic rate of the host, and the form in which the drug is delivered. Therefore, further research involving assessment of the anti-PEDV activity of MAG in vivo is required.

In conclusion, MAG exhibits anti-PEDV activity in vitro, with IC_50_ and CC_50_ values of 28.21 μM and 57.28 μM, respectively. Our data also demonstrate that MAG impedes viral replication, and that inhibition of viral proliferation is strongest when the host cells are exposed to MAG and the virus simultaneously.

## Data Availability

Not applicable.
